# The development and validation of the teacher professional identity scale in a Chinese university context

**DOI:** 10.1371/journal.pone.0293156

**Published:** 2024-03-06

**Authors:** Jie Zeng, Weijia Liu

**Affiliations:** 1 School of Foreign Languages, Chengdu Normal University, Chengdu, China; 2 Centre for Southeast Asian Economic and Cultural Studies, Chengdu Normal University, Chengdu, China; 3 Faculty of Languages and Linguistics, Universiti Malaya, Kuala Lumpur, Malaysia; National University of Modern Languages, PAKISTAN

## Abstract

Professional identity has become a central topic in teacher education research and a crucial factor in shaping teachers’ self-perception and perspectives on various aspects of their profession, including teacher roles, scholarly research, curriculum design, classroom instruction, instructional methods, and strategies, as well as their interactions within the educational context. Despite the considerable scholarly interest in teacher identity development, relatively few studies have considered how to measure teacher professional identity. This study developed and validated a new measurement of professional identity among Chinese pre-service teachers from an English language education program. A total of 560 pre-service teachers majoring in English language education were invited to participate in a survey and 542 questionnaires were deemed valid and subjected to analysis. Through this analysis, a scale with 17 items was developed, focusing on three different dimensions: professional self-efficacy, career commitment, and professional knowledge. After excluding items with a relatively poor correlation with factor loading, the final scale consisted of 13 items. The results showed that the developed scale has relatively good reliability (α = 0.939) and structural validity (χ^2^/df = 2.46, *p* < .001, CFI = 0.978, TLI = 0.971, SRMR = 0.033, RMSEA (90% CI) = 0.071 0.054, 0.089). This study may provide a quantitative instrument for future research to measure professional identity among pre-service teachers, both in Chinese and other contexts.

## 1. Introduction

Over the past two decades, research on teacher professional identity (TPI) has mushroomed in the broad field of teacher education [1]. Previous studies have revealed the crucial role of teacher identity in professional development and training: the sense of TPI could considerably influence the beliefs and practices related to teaching [2, 3]. Thus, facilitating PI can promote teacher development [4]. In this sense, a better understanding of TPI formation has theoretical and practical implications for teacher education research and professional development [5–7]. The literature on TPI has conceptualized it as a multifaceted process [5, 6]. The formation of TPI is subject to social interactions across time and space, shaped by various factors, for instance, experience, self-image, agency, and context [4]. Due to this complexity, previous studies have mainly adopted qualitative methods such as ethnographies to investigate TPI formation [e.g., 5, 7–9]. For example, Lopes and Pereira [3] relied on data collected from three sources [i.e., biographical narratives, documents, and interviews] at four points in time. This study has methodological merits in that multiple data sources were triangulated to explore identity formation among pre-service teachers. Similarly, Dimitrieska [10] used ethnographic methods of inquiry, such as interviews and observations, as a part of a case study research design. This study explored the interplay between teacher cognition and learning in forming teacher identity through the case analysis of a novice language instructor. However, most prior studies have employed qualitative research methods to examine specific elements that influence teacher identity. However, given the diverse range of factors that impact teacher identity, quantitative research can provide a means to measure and determine the most significant influencing factor. Furthermore, while there exists a substantial body of research pertaining to professional identity, limited attention has been given to the development of measurement tools for assessing teachers’ professional identity [11]. Moreover, the scarcity of studies examining the professional identity of novice instructors is particularly noteworthy. Considering China’s substantial teacher population, the establishment of specialized scales within the Chinese context holds significant research implications for the professional identity of teachers, the implementation of teacher education, and the creation of policies. Consequently, the primary objective of this study is to delve deeper into the professional identity of teachers. Furthermore, it aims to create and authenticate a novel measurement approach for assessing the professional identity of pre-service teachers in China.

## 2. Related literature

Both the personal and the contextual have been taken into consideration in recent studies on TPI [12, 13]. Without contextual factors or professional backgrounds, there is neither a self nor a PI; rather, the self is created via the process of meaningful and complicated social interaction [14]. The fact that the concept is multifaceted further complicates the process of developing a teacher’s PI [7, 14]. Despite this, given its significance, educational scholars have identified a range of characteristics that affect the development of a teacher’s PI, such as formal teacher preparation or field teaching experience [9, 12, 14, 15].

Previously mentioned studies provided deep insights into teacher identity, however, most of such studies prefer qualitative approaches [e.g., 12, 13]. Qualitative studies are not without limitations as they often require considerable time in data collection and analysis. This time-consuming nature constrains qualitative studies to a particular context and a relatively small sample of participants. In addition, the findings from qualitative research are not generalizable to a broader context, which makes the findings hardly comparable across studies. For the results of studies to be consistent, there is a need for quantitative studies that involve a large sample of participants across contexts. Studies of this type call for reliable instruments that allow data collection at a low cost. Questionnaires suit this particular need for convenience and low cost [16].

However, questionnaires have been lacking to measure teachers’ professional identity. The existing scales to measure professional identity are often related to other areas. For example, Bennett [17] utilized six different measures to measure the PI of newly graduated marketing students. The data collected in the study mainly comes from the students’ self-reported perceived membership to the profession (e.g., “I feel strongly that I am a member of the marketing profession.”) Tomlinson and Jackson [18] measured higher education majors’ emerging PI based on their responses to eight questions. These questionnaires were not assessed for their reliability and validity. In contrast, Tan et al. [19] developed and validated a professional identity scale with five factors. While they established the multi-dimensionality of the scale, the factorial structure and the interaction between the dimensions were not fully considered. Moreover, the scale is a general scale that measures the professional identity of college graduates. A scale that specifically measures the professional identity of preservice teachers is desired.

### 2.1 The contextual nature of professional identity

Researchers have argued that personal and contextual aspects should be considered when developing TPI scales [4, 20]. Reynolds [21] highlights that a person’s identity as a teacher is significantly influenced by their surroundings, the expectations of others, and the degree to which they allow these factors to shape them. Moreover, research findings indicate that becoming a teacher involves more than identifying oneself as a teacher but also being recognized by others and identifying with the communities of practice [22]. Most studies on TPI focus mainly on the inward or private facet of identity building [4, 23]. Since an individual’s personality traits are shaped by their social environment [24], the contextual factor merits attention in understanding identity development [4].

### 2.2 The Chinese context

The Chinese context is in striking contrast with the Western countries. In the first place, the social status of teachers has long been valued in Chinese society. For example, according to a recent survey by Dolton and Marcenaro [25], the social status of teachers is generally at the middle of the social rank among all the countries surveyed except China, Israel, and Brazil. Teachers in China were shown to be at the top of the social rank, whereas teachers in Israel and Brazil were at the bottom. It has also been indicated that teachers are more respected in China than in European countries and that about 50% of Chinese families support their family members to pursue a teaching career, compared to less than 8% of parents in Israel and Russia. It seems clear that an individual’s decision to pursue a teaching profession is subject to social culture and their parents’ opinions [26].

There are also practical considerations in pursuit of a teaching position. First, the teaching profession has been considered a stable and permanent job, especially for those in the public school system. Career stability and generous welfare benefits (often in those developed areas) attract thousands of university students to the job-seeking competition. The recent bleak job market makes teaching positions in public schools even more popular. Second, all children must receive an education. The teaching profession has become crucial in society [27]. Third, the public generally perceives the working environment for teachers as positive. Public school teachers are respected by students and their parents and have long summer and winter vacations. These discussions point to the presence of strong external influence on the decision to enter the teaching profession [28], which partly explains the growing number of students enrolled in the teacher education program [29].

Despite the strong influence of the social environment, it appears that Chinese students from teacher education programs do not necessarily take the teaching profession as their priority in career choice [9]. Student teachers may have a relatively weak commitment to teaching after they enter the teacher education program. The marked contrast between the external influence and the inward demotivation of Chinese student teachers raises the need to understand their professional identity development. However, few studies have considered the professional identity of pre-service teachers in the Chinese context.

The present study addresses this research gap by developing a scale of the professional identity of pre-service teachers in the Chinese context. The scale to be developed is expected to reflect the sociocultural difference [30, 31]. The results of the study can further the line of inquiry into teacher professional identity [8, 9, 32].

## 3. Method

### 3.1 Context and participants

The research was conducted in accordance with the Helsinki Declaration and the American Psychological Association (APA) code of ethics. The Academic Ethics Committee of the School of Foreign Languages at Chengdu Normal University approved the study (SFLRA-2022008). This study recruited adult college students from English education major at a public university in South-Western China beginning on October 15, 2022, and ending on December 15, 2022. This period corresponds to the commencement of classes in Chinese educational institutions, during which participants engage in third-year teaching theory and practice coursework and fourth-year practical training at their respective schools. This facilitates the centralization and convenience of data collection. Furthermore, the authenticity of the participants’ comments is enhanced by their presence in the genuine classroom setting. Participants were invited to participate in the questionnaire according to their own wishes and were informed of their right to withdraw from the study at any time. No personal information was disclosed before, during, or after this study. Students from an undergraduate program in English education at a public university in South-Western China were approached for the present study through purposeful sampling. All participants provided written informed consent before they decided to take part in this study. The program was intended to train English school teachers. The data participants in this study are comprised of all senior university students (n = 355) and juniors (n = 205) in the School of Foreign Languages at a Chinese university. On average, the participants were 21.1 years old. In accordance with research objective two, the investigation pertaining to data analysis employs the utilization of the structural equation model, which necessitates a somewhat substantial sample size of data, typically exceeding 500. To enhance the persuasiveness of the research findings, the researchers endeavor to maximize the data sample size by collecting a substantial number of samples. Ultimately, via the support of participants and their enthusiastic engagement, a cumulative count of 560 sample subjects was attained, which is excluding invalid responses (e.g., straight-lining). Furthermore, within the sample of 560 individuals, there were 502 females (almost ninety percent) and 58 males. The show of this gender ratio proves to be highly significant in the exploration of the professional identity of new teachers inside the Chinese educational framework. In the context of China, the teaching profession is widely seen as a secure and well-compensated vocation. Furthermore, it is noteworthy that teachers are granted numerous holidays, aligning with the societal norms in Chinese households that place an emphasis on women assuming the responsibility of familial caretaking. Furthermore, it is noteworthy that educators hold a significantly positive position within Chinese culture, with a considerable number of parents expressing a strong desire for their offspring to actively participate in the realm of education. The aforementioned factors have resulted in a discernible disparity in the male-to-female ratio within the teaching faculty in China. Representatively, the significant feedback from female pre-service teachers can be used to check the validation of the scales under investigation, especially in the Chinese context.

### 3.2 Instrument

This study draws on existing definitions and measures of professional identity. Various attempts have been made to define professional identity. For example, Ibarra [33] defined professional identity as “the relatively stable and enduring constellation of attributes, beliefs, values, motives, and experiences in terms of which people define themselves in a professional role” [p. 764]. Tan et al. [19] defined professional identity as “the self that has been developed with the commitment to perform competently and legitimately in the context of the profession” [p. 1505]. Tan conceptualized professional identity as having five dimensions: (1) Knowledge about professional practices, (2) Experience with the profession, (3) Having the professional as a role model, (4) Professional self-efficacy, and (5) Preference for a particular profession.

This study defines professional identity as the beliefs and values to perform in the context of a profession. The study has adapted the Professional Identity Five-Factor Scale developed by Tan et al. [19], which has been widely used in previous studies, especially in Chinese TPI research. This study includes two constructs from Tan et al. [19]: knowledge about a profession and professional self-efficacy. The construct of experience with the profession is more about factual information than perception, and having a role model is more of a predictor of PI than of itself [33], thus it is not suitable for measuring TPI of pre-service teachers because they lack experience in genuine teaching contexts. The construct of preference for a profession was not operationalized in a Likert scale format. Taking into account the particularity of China’s teacher education (e.g., the existence of both public and self-funded, primary and secondary school internships, matching education assistance to poor and remote areas, more than 2,000 years of teacher education culture, etc.) This study developed a new measure named professional commitment, which is claimed to be one of the most important elements in the professional identity of Chinese teachers in the academic circle of teacher identity research. Six items respectively were formulated to measure professional self-efficacy and professional commitment, and five were formulated for knowledge about the profession. These items were formulated based on existing items or definitions from the literature on professional identity. The scale initially consisted of 17 items.

The scale was included in a questionnaire along with a set of questions about the participants’ demographic information. The participants responded to the questions on a 5-point Likert scale.

### 3.3 Data collection and analysis

The questionnaire was administered to undergraduate students majoring in English education. The questionnaire was administered through an online platform. The link to the questionnaire was sent to students from the undergraduate program. The data were first screened to remove invalid responses (e.g., straight-lining). The dataset used for this study included 542 valid responses from pre-service teachers.

The scale development and validation were completed in four steps: (1) Split the data set into two samples; (2) Perform Principal Component Analysis (PCA) to establish the factor structure; (3) Select items for the scale; (4) Confirm the structure of the developed scale using Confirmatory Factor Analysis [CFA].

## 4. Results

### 4.1 Step 1: Split the data

The data set was randomly split into two samples (*n* = 271 and *n* = 289) using SPSS 17 (see Table 1). Sample 1 was used for item selection and factor structure examination, whereas Sample 2 was used to assess the validity and reliability of the resultant scale.

**Table 1:** Demographic information for Samples 1 and 2.

	Sample 1	Sample 2
Sample size	271	289
Gender	90.8% female	87.4% female
Age	*M* = 20.95 (*SD* = 1.08)	*M* = 21.07 (*SD* = 1.05)

### 4.2 Step 2: Establishing the factor structure

The items formulated for the scale were first examined for their correlations with their hypothesized dimensions. The items were examined based on a range of indices (i.e., Corrected Item-Total Correlation, Cronbach’s Alpha if Item Deleted, Communalities, and Factor loadings). Three items (i.e., KPP 5, PS6, and PC1) were removed from the item pool due to their relatively low correlation or factor loadings.

The dataset on the remaining 13 items was analyzed via Principal Component Analysis (PCA) with an oblique promax rotation. Promax was chosen because correlation was assumed among the three factors in the scale. Bartlett’s test of sphericity was significant, χ^2^(91) = 3381.60, *p* < .001, and therefore indicated that the items of the proposed scale are related and factor analysis would be a valuable method to pursue. The Kaiser-Meyer-Olkin Measure of Sampling Adequacy result (KMO = .941) further confirmed the suitability of the data for structure detection (KMO > .80; Field, 2005). In line with the theoretical model, which included three different dimensions: professional self-efficacy, career commitment, and professional knowledge, we decided to fix the number of factors to 3 in the extraction. The PCA analysis suggested that the scale’s factor structure was in line with the theoretical model, with most of the formulated items loaded into their hypothesized dimensions (see Table 2).

**Table 2:** Factor loadings of the final scale.

Items		F1	F2	F3
PS4	I believe I can do well in my teaching career.	.975		
PS5	I have no doubt that I can master all the skills for the teaching profession.	.875		
PS1	I believe I can be professional in my career as a teacher.	.798		
PS2	I am well prepared for the teaching profession.	.791		
PS3	I believe I can easily get along, cooperate, and chat with my future colleagues.	.780		
KPP3	I have a good understanding of the role and responsibilities of a teacher.		.933	
KPP2	I know the kind of people I want to work with in the future.		.919	
KPP4	I know what apps, tools, and devices to use as a teacher.		.913	
KPP1	I understand the nature of the teaching profession.		.895	
PC6	I hope to be a teacher in the future.			.959
PC3	It is important for me to be a member of the teaching profession.			.907
PC5	I share the values of the teaching profession.			.735
PC2	I can positively identify with people in the teaching profession.			.678

*Note*. F1 = Professional self-efficacy; F2 = Knowledge about the profession; F3 = Professional commitment.

### 4.3 Step 3: Select items for the scale

Items that loaded into a dimension but were not initially formulated for that dimension were removed for inconsistency with theory. The factors of PS and KPP both included four items (i.e., PS1, PS2, PS3, PS4, and PS5 for PS, and KPP1, KPP2, KPP3, and KPP4 for KPP). While the results indicated that four items clustered for the factor of PC, the loadings of two factors (.735 for PC5 and .678 for PC2) were qualitatively lower than the other two (.959 for PC6 and .907 for PC3). The factor loadings of the final scale show that the 13 items selected for the TPI measurement are valid and reliable.

### 4.4 Step 4: Confirm the factor structure

**Fig 1:**
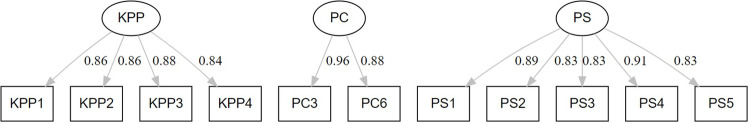
Model 1: The first-order model.

Two CFA models were tested to confirm the construct validity. The first was a three-factor first-order CFA model. The results of this first-order CFA model showed acceptable model fit χ^2^ (41) = 100.68; χ^2^/df = 2.46; *p* < .001; CFI = 0.978; TLI = 0.971; SRMR = 0.033; RMSEA (90% CI) = 0.071 [0.054, 0.089] (see Fig 1).

The second was a three-factor second-order model. The results showed that the second-order model had a relatively poorer model fix χ^2^ (34) = 92.83; χ^2^/df = 2.730; *p* < .001; CFI = 0.976; TLI = 0.968; SRMR = 0.107; RMSEA (90% CI) = 0.077 [0.059, 0.096].

The statistics show that in the first-order and second-order models, their CFI > 0.9, TLI > 0.9, RMSEA < 0.08, which means that both CFA models have a high degree of fit. The SRMR of the first-order model is less than 0.05, while the SRMR of the second-order model is greater than 0.05. When comparing the values of the first-order model and the second-order model, it is obvious that the model fit reflected by the values of the first-order model is better than that of the second-order model.

## 5. Discussion

This study seeks to develop and validate a scale for professional identity development among pre-service teachers. Factor analysis showed that the scale has relatively good reliability and validity. This newly developed scale can serve as an instrument to measure professional identity for future teacher education and professional development research.

This study contributes new insights into the teacher professional identity research topic. Theoretically, first, it is one of the few studies that seek to develop a measure of pre-service teachers’ professional identity in the context of Chinese universities. While China is one of the top three contexts that receive research attention to teachers’ identities [34], a measure of teacher professional identity has been overdue in the past few decades. Given the sociocultural differences between Western countries and China, the scale developed in the Chinese context can better reflect the professional identity of pre-service teachers in the local context. Second, this study provides a reliable measure for future research on teacher professional identity. In addition to qualitative interviews and reflective journals, future research may solicit responses using a quantitative measure, which fills the gap of the research instrument. Multiple data sources can contribute more insights into the complicated phenomenon of teacher identity formation.

Practically, first, the scale can also be used in interventions or metacognitive instructions to raise teacher trainees’ awareness of professional identity. Besides, the scale has the potential to enhance the motivation of pre-service teachers to engage in the act of teaching. By utilizing the scale, pre-service teachers can engage in a comprehensive evaluation and examination of their personal perspectives and attitudes toward the educational landscape. This measure has the potential to enhance the Chinese teacher education system by facilitating the identification of educators who possess a genuine passion for the field of education, thus contributing to the overall improvement of teaching quality. Furthermore, the scale incorporates the distinctive context in which instructors in China operate. The integration of teacher education content with the findings derived from the scale and then adjusting some teacher training strategies can enhance the efficacy of teaching methodologies and tactics, which, in turn, train pre-service teachers with the necessary skills to effectively address the career challenges prevalent in China. In addition, the elements that influence the building of teacher identity, as shown in this scale, can be sensible for Chinese education policymakers. By recognizing certain factors, policymakers can better address the requirements of pre-service teachers and develop policies that are more rational and supportive in promoting the construction of teacher identity. Furthermore, the further update of education policy has the potential to enhance the level of satisfaction among pre-service teachers. This, in turn, can serve as a catalyst for enticing highly qualified graduates to pursue careers in the field of education and subsequently contribute to the retention of talented individuals within China’s educational sector.

## 6. Conclusion

China has the largest number of teachers of any country in the world. The teaching profession is highly attractive to college students, whether they are graduates of teaching schools or not. It is very important to develop a reasonable test scale based on both qualitative and quantitative research of TPI. As a supplement to the previous qualitative research on teacher identity, our study shows that theoretically, teacher identity is not limited to the subjective statements of pre-service teachers, but can also be measured by a specific scale, which provides a new theoretical approach for TPI research. The TPI scale developed and validated in this study may serve as an effective measurement tool used to assess and quantify the professional identity of pre-service teachers in the Chinese context. It also provides a structured and standardized instrument for measuring and understanding the beliefs, values, attitudes, and perceptions that pre-service teachers have about their professional role and identity. Cultivating pre-service teachers’ TPI according to the result of the scale test of this study may achieve the following effects:

(1) Enhancing teacher professional development: The TPI scale can stimulate pre-service teachers’ willingness to teach and lead them to become qualified teachers. By offering a framework to evaluate and comprehend one’s PI, the scale may aid in the professional growth of teachers. It gives pre-service teachers the chance to consider their attitudes, values, and ideas about education, which can help them continue to develop as qualified teachers.
(2) Improving teaching quality: Teaching methods and instructional approaches are directly influenced by a teacher’s PI. It is possible to pinpoint the distinctive components that influence TPI in China by creating a scale tailored to the local culture. The effectiveness of teaching and learning in the classroom can be improved by implementing focused professional development initiatives and programs that are in line with the unique needs and cultural characteristics of Chinese teachers.
(3) Tailoring support and resources: A TPI scale may provide policymakers, educational institutions, and teacher preparation programs with information on the particular difficulties and needs of Chinese teachers. It enables the creation of specialized policies, resources, and support systems that are tailored to the requirements and goals of Chinese educators in terms of their careers. By doing this, it is made sure that the assistance offered fits with their identity, cultural beliefs, and educational environment.
(4) Promoting teacher well-being: Teachers who are satisfied and feel well-cared for work better. Teachers’ motivation, work satisfaction, and general well-being all improve when they have a strong sense of their PI and feel appreciated in their field. The creation of a TPI scale can aid in determining the elements that contribute to teachers’ well-being, improving work satisfaction, and reducing burnout.
(5) Promoting collaboration and networking: The TPI scale offers a common language and structure for teachers to engage in professional discourse, cooperation, and networking. It encourages pre-service teachers to share their expertise, intern experiences, and teaching methods so they may grow as professionals and learn from one another. The sharing of best practices, innovation, and professional development is encouraged in this cooperative atmosphere.

Using the current TPI scale, researchers, educators, and policymakers may be able to better understand the elements that influence teacher identity and how it affects instructional strategies, professional growth, and overall job satisfaction. By employing the scale, researchers and educators can quantitatively analyze the responses and produce data that can be used to shape professional development programs, inform policy decisions, and comprehend the connection between teacher identity and various outcomes, including teaching effectiveness, job satisfaction, and student achievement. In the meantime, it is worth noting that several scales might exist, as they are created by researchers and subject-matter specialists depending on their particular goals and research concerns. Compared with our scale, those scales may vary in the number of items, response options, and dimensions they assess, but the underlying goal remains to provide a standardized way of measuring TPI.

## 7. Research limitations

This study is not without limitations. First, the sample used for scale development was limited regarding the sample size and disciplines involved. Also, the sample included a predominant proportion of female respondents. A larger sample of pre-service teachers from various disciplines and institutions could help increase the generalizability of the findings across contexts. To enhance the applicability of future study outcomes across many contexts, it is recommended that future studies consider augmenting the sample size by including a larger cohort of preservice teachers from various disciplines and institutions. Furthermore, the acquisition of novel data can be facilitated by employing specialized techniques, including data translation, flipping, and zooming. In relation to the limitations imposed on teachers’ career choices based on gender, future research endeavors should adopt some measures to balance gender-related data, enhance the representation of male participants, and strive to achieve equilibrium in gender disparities. Second, the “Professional commitment” sub-scale now consists of two items. While it currently has relatively good reliability, it is not clear whether the reliability of the sub-scale is a result of the small number of indicators. New items are desirable for the sub-scale to increase its coverage and reliability. Various data sources, such as focus groups and in-depth interviews, can be exploited to develop new items and improve the theoretical constitutions of the scale. Finally, the concept of professional identity involves multiple dimensions. While this study has drawn on relevant literature and lengthy discussions with Chinese pre-service teachers, other aspects of professional identity could remain untouched. Future studies to refine this scale may administer this scale to a different sample of pre-service teachers and interview respondents with different levels of identity formation. Interview data makes it possible to identify hitherto unidentified aspects of teacher professional identity and factors determining teacher professional identity.

## Supporting information

S1 File Minimal data set. (RAR)
